# Effects of small-sided game training on lower limb explosive strength in handball players: a single-arm meta-analysis

**DOI:** 10.3389/fspor.2024.1477347

**Published:** 2024-11-25

**Authors:** Ran Wang, Quanzhi Li, Weiqi Xue

**Affiliations:** ^1^ Sports Department, Nanjing University of Science and Technology, Nanjing, Jiangsu, China; ^2^ Sports Training Academy, Nanjing Institute of Physical Education and Sports, Nanjing, Jiangsu, China; ^3^ Sports Department, Nanjing Agricultural University, Nanjing, Jiangsu, China

**Keywords:** SSG, handball, CMJ height, 20-m (s), meta-analysis

## Abstract

**Objective:**

This meta-analysis systematically evaluates the influence of small-sided game (SSG) on the counter-movement jump (CMJ) height and 20-meter sprint capabilities of handball players.

**Methods:**

Systematic searches of PubMed, Web of Science, Cochrane Library, and China National Knowledge Infrastructure(CNKI) databases were performed up to February 2024.

**Results:**

A total of 8 studies with 184 participants were included. Meta-analysis demonstrated significant improvements in CMJ (cm) in handball players (Weighted Mean Difference (WMD) = −1.06, 95% CI[−1.99, −0.12], Z = 2.22, *P* = 0.03). For the 20 m sprint capability, the results were WMD = −0.07, 95% CI[−0.14, 0.01], Z = 1.82, *P* = 0.07. Subgroup analysis by age: ≥19 years old WMD = −0.02, 95% CI[−0.08, 0.03], and <19 years old WMD = −0.10, 95% CI[−0.21, 0.00], indicating no significant impact on the 20-m (s) performance improvement. Gender subgroup analysis showed male WMD = −0.01, 95% CI[−0.08,0.07] and female WMD = −0.11, 95%CI[−0.19, −0.03; *P* < 0.05], suggesting better improvement in females.

**Conclusion:**

The present study reveals that SSG training has varying impacts on 20 m sprint performance among handball players of different ages and genders. Specifically, there is no significant improvement in the 20 m sprint performance between players aged <19 and ≥19, while female players show greater improvement in the 20-m (s) compared to male players. These differences could be attributed to the physiological, psychological, and adaptive training differences between athletes of different ages and genders. Although SSG plays an essential role in training handball players, particularly in enhancing lower limb explosive strength and overall game performance, it is recommended to combine SSG with other targeted strength and explosive power training to maximize the enhancement of lower limb explosive power in handball players. Comprehensive training can effectively improve the lower limb explosive strength of athletes while also addressing the development of other key athletic qualities to achieve the best training outcomes. Therefore, coaches should fully consider the individual differences and training needs of athletes when designing training plans, and reasonably allocate the proportion and sequence of SSG with other training methods to maximize training effectiveness.

## Introduction

Handball is a high-intensity offensive and defensive sport consisting of pushing, tackling, jumping, sprinting, blocking, shooting (1), and stealing actions (2), requiring players to compete for 60 min on a 20m × 40 m court. The game involves a transition from the formerly popular 11-player outdoor version to the 7-player indoor version since 1964 and has since achieved international standardization (3). The sport incorporates both high-intensity collisions and lower intensity sliding (4), and walking actions (5), exemplifying a typical same-field contact sport.

SSG is a method of training conducted on a smaller pitch, usually involving fewer players. This training method mimics the dynamics of actual gameplay, allowing coaches to adjust training content based on specific objectives, such as inducing physiological/physical stimuli or developing technical and tactical behaviors (6). The application of SSGs in handball training has been widely researched and practiced, demonstrating significant effects on improving athletes’ physical performance and game performance. In small-sided handball games, frequent physical contact drives the fatigue mechanism, which differs between the upper and lower limbs (1, 7–9). Studies have shown that physical contact can lead to short-term impairment in motor performance due to the neuro-muscular overload of the upper body, with the lower limbs typically involved in low strength, high speed movements, while the upper body performs high strength, low speed actions. Studies have indicated (10) that handball demands a high level of lower limb explosive power from athletes. During handball matches, athletes frequently need to perform rapid movements, jumps, and quick direction changes, all of which rely on strong lower limb explosive power. This is widely acknowledged. However, scholars both domestically and internationally have not reached a consensus on the effectiveness of small-sided game training in enhancing the lower limb explosive power of handball players. Hammami (5), in a study on the effects of resistance band training and SSG on the physical performance of female handball players, found significant improvements in the 20 m sprint time (*P* < 0.001). However, Bělka (11) found no significant difference (*P* > 0.05) in the 20 m sprint time between the experimental group and the endurance training group in female handball players. Similarly, Dello Iacono (8) reported significant changes (*P* < 0.001) in CMJ performance among female handball players with body contact in SSG. In contrast, Buchheit found no significant change in CMJ performance with SSG among young elite athletes.

The 20 m sprint test is a simple and direct method to assess speed and power. It measures an athlete's maximum speed over a short distance, crucial for sports requiring rapid acceleration and explosive power. Studies indicate a significant correlation between 20 m sprint times and lower limb explosive power (10, 12). Similarly, the CMJ test assesses lower limb explosive power by measuring the maximum height an athlete can reach during a CMJ. The CMJ test evaluates muscle strength, neuromuscular coordination, and energy conversion efficiency, with studies showing a high correlation between CMJ performance and lower limb explosive power (13, 14).Therefore, this study employs the internationally recognized lower limb explosive strength evaluation indicators: CMJ (15) and 20 m short-distance sprint (16). The primary aim of this research is to assess the effect of SSG on the lower limb explosive power of handball players through a meta-analysis. By systematically collecting and analyzing relevant literature, this study aims to provide a comprehensive understanding of the most effective training methods for enhancing the lower limb explosive power of handball players, thereby offering a scientific basis for coaches and athletes to select the most suitable training methods.

### Research methods

This study adhered strictly to the PRISMA guidelines for meta-analyses (17).

### Literature search

Literature searches were conducted using the PubMed, Web of Science, Cochrane Library, and China National Knowledge Infrastructure (CNKI) databases. Search terms in Chinese included: small-sided games (training), handball players, athletic performance, sports results, explosive power, CMJ, and short-distance sprinting ability, among others. English search terms included: small-sided games, SSG, handball player, performance, CMJ, sprinting ability, and lower extremity explosive power. Searches were conducted using combinations of two or more terms, with the search cut-off date being February 1, 2024 (Table 1).

**Table 1:** Literature search strategy.

Database	Search terms
PubMed	#1 “small-sided games”[Mesh] OR SSG [Title/Abstract] OR SSG training, Small-sided game [Title/Abstract] OR ssg training [Title/Abstract] OR Ssg training [Title/Abstract] OR handball player [Title/Abstract] OR D handball [Title/Abstract]. #2 “Lower extremity explosive power” [Mesh] OR Sprinting ability [Title/Abstract] OR CMJ [Title/Abstract] OR 20 m [Title/Abstract] OR performance [Title/Abstract] OR explosive power [Title/Abstract]. #3 1 AND 2.
Cochrane Library	#1 Mesh: [small-sided games] explode all trees. #2 Mesh: [Lower extremity explosive power] explode all trees. #3 (Small-sided games): Title/Abstract/Key Words. #4 (SSG): Title/Abstract/Key Words. #5 (SSG training): Title/Abstract/Key Words. #6 (Ssg training): Title/Abstract/Key Words. #7 (ssg training): Title/Abstract/Key Words. #8 (Lower extremity explosive power): Title/Abstract/Key Words. #9 (Sprinting ability): Title/Abstract/Key Words. #10 (CMJ): Title/Abstract/Key Words. #11 (20 m): Title/Abstract/Key Words. #12 (performance): Title/Abstract/Key Words. #13 (handball player): Title/Abstract/Key Words. #16 #1 OR #3 OR #4 OR #5 OR #6 OR #7. #17 #2 OR #8 OR #9OR #10OR #11OR #12 OR #13. #18 #16 OR #17.
CNKI	#1 Mesh: small-sided games (Small-sided games). #2 Mesh: Lower extremity explosive power. #3 #1 and #2 #4Title/Abstract/Key Words: SSG training (SSG). #5Title/Abstract/Key Words: CMJ or 20 m or Lower extremity explosive power or performance or handball player. #6 #4 and #5.
Web of Science	#1Mesh: SSG training, small-sided games, Small-sided game, Ssg training, SSG, ssg training. #2 Mesh: Lower extremity explosive power. #3 #1 and #2 #4Title: SSG training, small-sided games, Small-sided game, Ssg training, SSG, ssg training #5 Title: Lower extremity explosive power OR Sprinting ability OR CMJ OR 20 m OR handball player OR performance. #6 #4 and #5. #7 Abstract: T SSG training, small-sided games, Small-sided game, Ssg training, SSG, ssg training. #8 Abstract: Lower extremity explosive power OR Sprinting ability OR CMJ OR 20 m OR handball player OR performance. #9 #7and#8.

### Inclusion and exclusion criteria

Inclusion and exclusion criteria were established based on the “PICOS” principle (17).

Inclusion criteria:

 1. Subjects were experienced handball players without any history of injuries;
 2. Intervention measures for the experimental group included SSG, while the control group underwent high-intensity interval training, specialized skill training, or traditional physical training;
 3. The experimental design was a randomized control trial without any single-blind or double-blind restrictions;
 4. Outcome measures included at least one indicator affecting lower limb explosive power such as CMJ or 20-m(s) short-distance sprinting ability;
 5. The study type was based on outcome measures derived from pre- and post-experiment data from a single trial.

Exclusion criteria:

Inability to access full text; duplicate publications; non-handball SSG interventions; non-randomized control trial designs; lack of necessary experimental data, such as means, standard deviations, outcome indicators, sample information, etc.

### Literature screening

Initially, two reviewers conducted searches for Chinese and English literature using the search terms from the “Literature Search” strategy. Subsequently, based on the “PICOS” principles, they screened titles and abstracts, conducted a preliminary review of the full texts, and downloaded relevant publications. Finally, remaining publications were thoroughly reviewed and discussed to determine the final set of included literature. In cases of disagreement between reviewers, a third reviewer was consulted, and a joint discussion determined the inclusion of the literature.

### Data extraction

Data from included publications were recorded in an Excel spreadsheet, which included: (1) authors and publication dates; (2) sample sizes and ages of study subjects; (3) intervention periods, frequency, and training programs of experimental designs; (4) outcome indicator data.

### Quality assessment of literature

The Cochrane Collaboration's “risk of bias tool” in Review Manager 5.4 software was used to assess the risk of bias in the literature. Assessment categories included random sequence generation, allocation concealment, implementation and assessment of blinding, completeness of outcome data, selective reporting, and other sources of bias. Outcomes were classified as “low risk of bias”, “high risk of bias”, or “unclear”. The results were presented visually in a risk of bias graph.

### Data analysis

Review Manager 5.4 software was used for statistical analysis of outcome indicators from included literature. Heterogeneity among studies was first assessed using the “I2” statistic. An I2 of 0 indicated no heterogeneity and complete homogeneity; an I2 < 50% indicated mild heterogeneity, analyzed using a fixed-effect model; an I2 between 50% and 75% indicated moderate heterogeneity, analyzed using a random-effects model; and an I2 > 75% indicated high heterogeneity, also analyzed using a random-effects model, with sensitivity and subgroup analyses conducted to explore potential sources of heterogeneity (18). When outcome indicators were continuous variables with uniform units, mean difference (MD) and 95% confidence interval (CI) were used for statistical analysis. Otherwise, the standardized mean difference (SMD) (19) was employed.

## Results

### Literature search results

**Figure 1:**
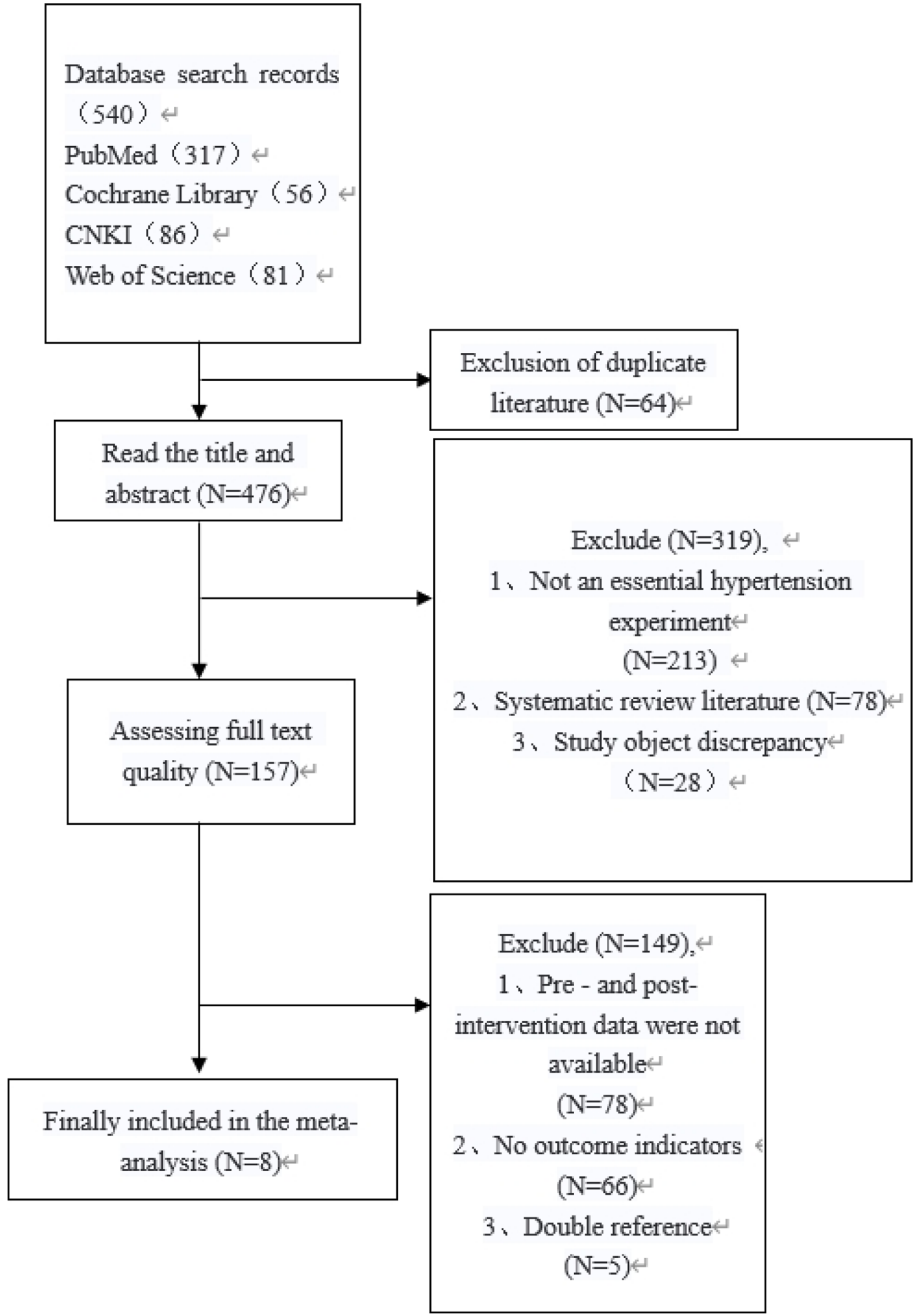
Literature screening flowchart.

According to the Literature Search Strategy, a total of 540 related articles were retrieved from four electronic databases: PubMed, Web of Science, Cochrane Library, and China National Knowledge Infrastructure (CNKI). Initially, 64 duplicate articles were excluded. Subsequently, 319 articles were excluded based on title and abstract review, including non-SSG intervention studies, systematic reviews, and irrelevant subjects. Reading the full texts and evaluating their quality, an additional 149 articles were excluded due to the lack of experimental data, outcome measures, or repeated citations. Finally, 8 articles were included in the meta-analysis. The literature screening process and results are presented in Figure 1.

### Basic characteristics of the included literature

The 8 articles included in this study were published between 2009 and 2023, encompassing a total of 184 subjects with no injuries and training experience. The intervention periods were 6 weeks or longer, with a frequency of at least twice a week. Training programs included SSG interventions in the experimental group, and routine handball training, high-intensity interval training, endurance training, and specialized training simulations in the control group, with SSG modifications involving changes in the number of players, rules, and court size (20). Outcome indicators included CMJ and 20-m short-distance sprints (Table 2).

**Table 2:** Basic characteristics of included literature.

Article	Sample size	Age	Intervention characteristics			Outcome
			Weeks	Frequency	Training scheme	
Bělka (11)	E: 9 C: 9	E: 21.22 ± 3.03years; C: 23.78 ± 3.77 years	6weeks	2 times/week	Regular Training: Combination of attack, defense, strength, speed, etc. In the last 40 min, SSG group 4 vs. 4 continuous 4 SSG, each time for 4 min, with a rest interval of 3 min. Participate in tournaments on two weekends. RG group for continuous endurance training, with an average heart rate intensity higher than 80% of HRmax.	20m
Buchheit (21)	E:15 C: 17	15.5 ± 0.9 years	10 weeks	2 times/week	Regular training: strength, technical tactics, etc. HBT group 4 vs. 4, 2 min 30 s to 4 min SSG, with 30 s rest. One formal match per week. HIIT group for 12–24 × 15s running, with 15s intervals.	CMJ
Dello Iacono (7)	E: 9 C: 9	24.8 ± 4.4 years	8 weeks	2 times/week	Regular small field training: strength, technical tactics. Weekend matches. RSS group two sets of 14–17 repeated 20-meter sprints and specific skills, with 20 s of recovery in between.	CMJ, 20m
Dello Iacono (8)	E: 12 C: 12	19.3 ± 0.4 years	8 weeks	7 times/week	Regular physical training. 5 sets of 20 × 30 m 3 vs. 3, with 1 min intervals. One formal match per week.	CMJ
Hammami (5)	E: 13 C: 13	15.8 ± 0.2 years	10 weeks	5 times/week	SSG group mainly for technical tactics and regular training, simulating matches. The experimental group uses elastic bands instead of specialized handball training.	CMJ, 20m
Iacono (1)	E: 9 C: 9	25.6 ± 0.5 years	8 weeks	2 times/week	SSG group 5 rounds of 3 min 40 × 20 m field 3 vs. 3 SSG, with 1 min intervals. HIIT includes 12–24 × 15 s of high-intensity running, with 15 s of recovery in between.	CMJ, 20m
Jurišić (22)	E: 12 C: 12	E: 16.06 ± 0.80 years C: 16.2 ± 1.28 years	8 weeks	4 times/week	SSG group combines technical, strength, and speed training twice a week. Two matches per week. HIIT group with ball possession warm-up, high-intensity running, interspersed with intervals.	CMJ, 20m
Mikalonytė (23)	E: 12 C: 12	16.2 ± 1.5 years	10 weeks	4 times/week	Regular technical and tactical training. 2 vs. 2, 3 vs. 3, 4 vs. 4 randomly but not repeated, each time for 10 min, with 1 min intervals. One formal match on the weekend. SMHT group conducts 3 rounds of 10 m handball matches, with a one-minute interval. All maintain a high pace.	CMJ, 20m

E, experimental group; C, control group; SSG group, small-sided game training group; RG, endurance control group; HBT, handball-specific training in small fields; HIIT, high-intensity interval training; RSS, repeated sprint shuttle ability group; SMAH, simulated official match group.

### Risk of bias in the literature

**Figure 2:**
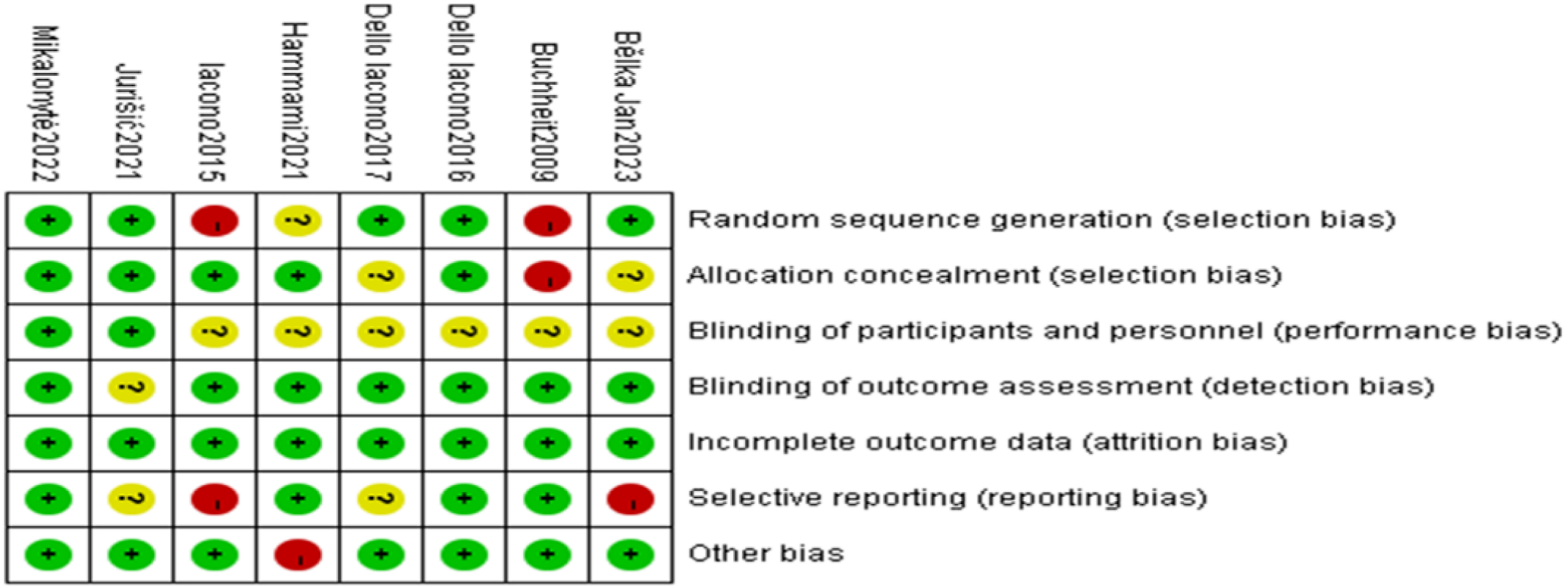
Literature bias risk assessment.

Two authors (RW and QZL) independently recorded the “Materials and Methods” sections of the 8 included studies. The methodological quality was assessed using Cochrane's risk of bias tool, including low risk, high risk, and unclear. Discrepancies were resolved through discussion with a third author (XWQ). Results were as follows: random sequence generation—5 studies rated low risk, 1 unclear, and 2 high risk; allocation concealment—5 low risk, 2 unclear, and 1 high risk; participant and personnel blinding—2 low risk, 6 unclear; outcome assessment blinding—7 low risk, 1 unclear; completeness of outcome data—8 low risk; selective reporting—4 low risk, 2 unclear, and 2 high risk; other biases—7 low risk, 1 high risk (Figure 2).

## Meta-analysis of small-sided game training

### CMJ

**Figure 3:**
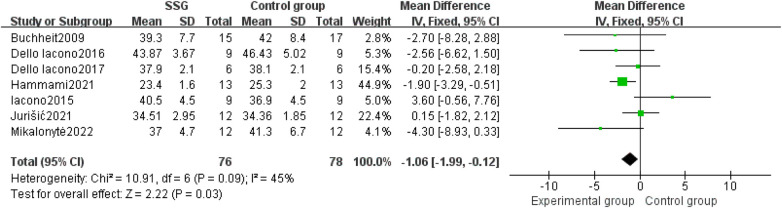
Forest plot of the effect of small-sided games training on handball players’ CMJ.

Seven articles contributing to a total of 154 subjects were included for the CMJ parameter. Meta-analysis results showed I^2^ = 45%, *P* = 0.09, indicating low heterogeneity, hence a fixed-effect model was chosen. The combined effect size WMD was −1.06 with a 95% CI of [−1.99, −0.12], indicating a significant statistical difference with Z = 2.22 and *P* = 0.03 < 0.05, confirming that small-sided game training has a positive impact on handball players’ CMJ performance (Figure 3).

### 20-m (s)

**Figure 4:**
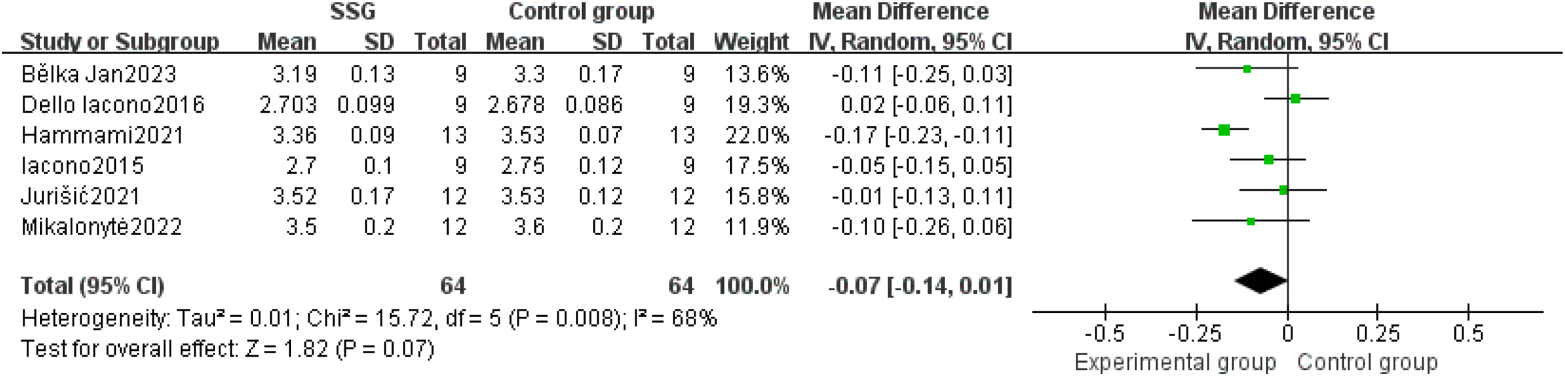
Forest plot of the effect of small-sided games training on handball players’ 20 m sprint ability.

Six articles comprising a total of 128 subjects were included for the 20 m sprint ability parameter. Meta-analysis results showed I^2^ = 68%, *P* = 0.008, reflecting high heterogeneity, leading to the selection of a random-effects model. The combined effect size WMD was −0.07 with a 95% CI of [−0.14, 0.01], Z = 1.82 and *P* = 0.07 > 0.05, indicating no significant statistical difference in the 20 m sprint results (Figure 4). Due to high heterogeneity, an initial “one study removed at a time” sensitivity analysis was conducted to explore potential sources of heterogeneity.

### Sensitivity analysis

Table 3 presents sensitivity analysis for study heterogeneity in 20 m sprinting ability using the “one study removed at a time” method. Removing Hammami (2021) resulted in a homogenous research landscape, but WMD did not show significant differences with *P* = 0.25 > 0.05. However, removing Dello Iacono (2016) led to a low heterogeneity I^2^ = 48% and a 95% CI of [−0.16, −0.03], with *P* = 0.005 < 0.05, indicating significant results and suggesting this article as a potential source of heterogeneity.

**Table 3:** WMD situation after removing individual studies.

Excluding studies	After excluding WMD and 95%CI value	After excluding I^2^	The *p*-value of I^2^ after excluding	The *p*-value of after excluding
Bělka Jan (11)	−0.06 [-0.15,0.02]	74%	0.004	0.16
DelloIacono (17)	−0.10 [−0.16, −0.03]	48%	0.1	0.005
Hammami (5)	−0.03 [−0.08, 0.02]	0	0.43	0.25
Iacono (1)	−0.07 [−0.16, 0.02]	74%	0.004	0.11
Jurišić (22)	−0.08 [−0.17, 0.00]	71%	0.007	0.06
Mikalonytė (23)	−0.10 [−0.26, 0.06]	75%	0.003	0.13

### Subgroup analysis

**Figure 5:**
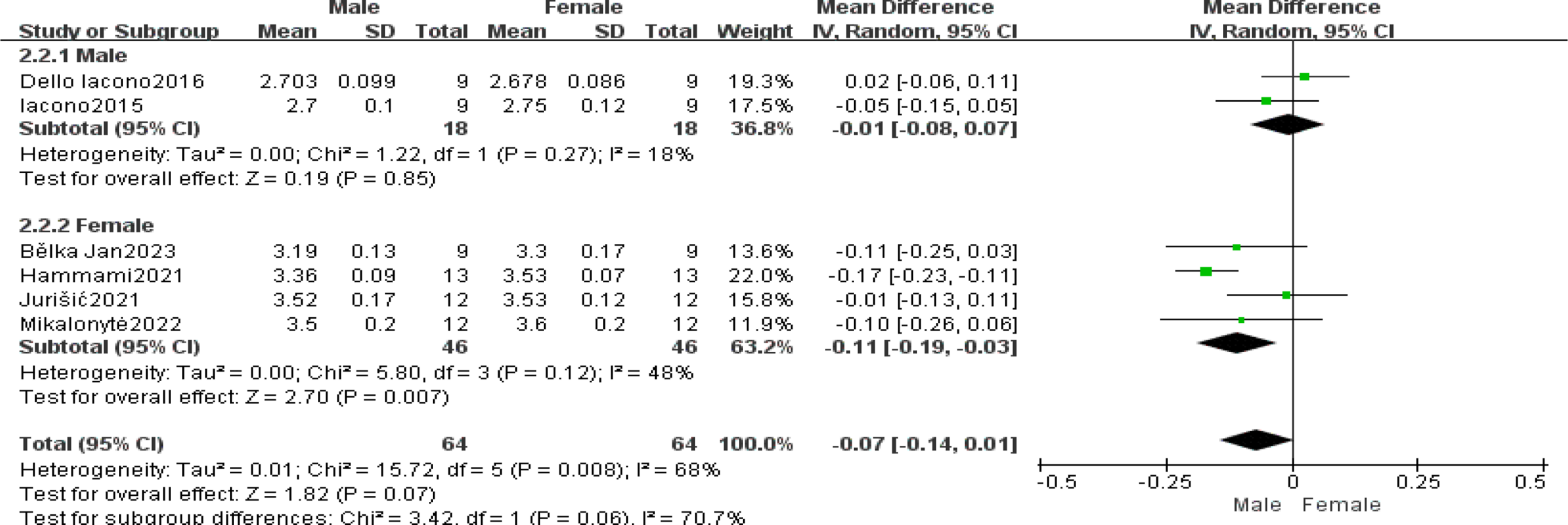
Subgroup analysis of 20 m sprint ability: gender.

**Figure 6:**
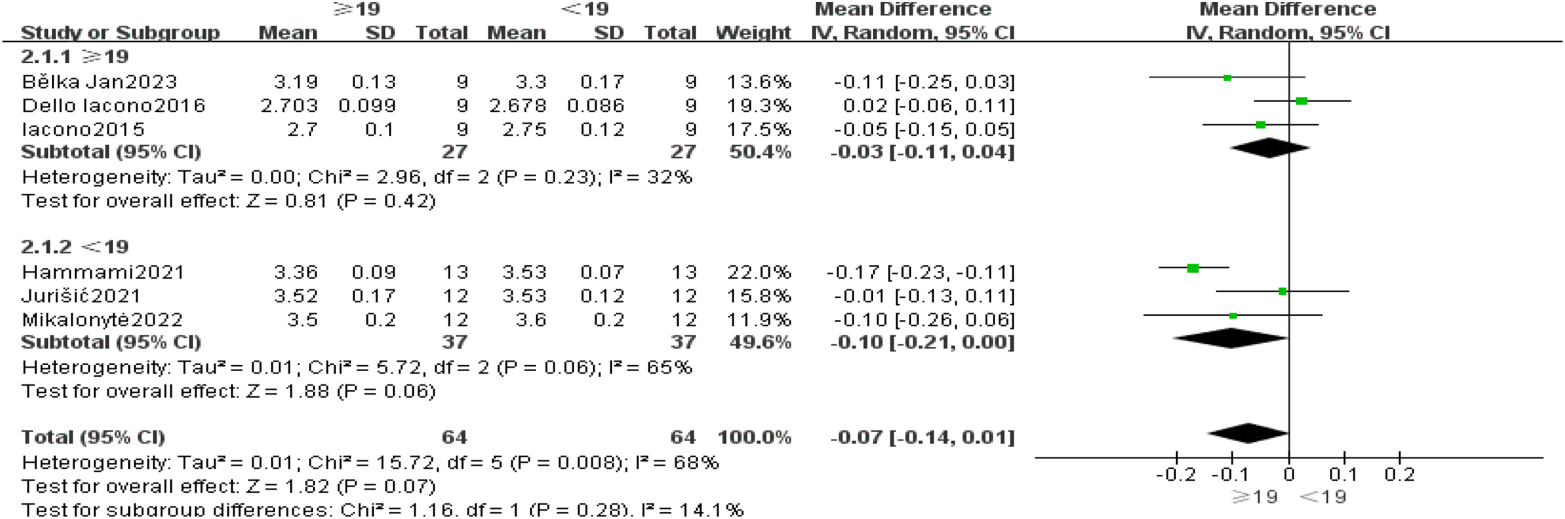
Subgroup analysis of 20 m Sprint Ability: Age.

Subgroup analysis was conducted based on potential heterogeneity sources, such as age and gender. Age was defined according to the World Health Organization's definition of adolescents: 10–19 years, aiming for alignment with selection criteria (21). Six articles differentiated handball players by gender, with 3 articles each for <19 and ≥19 years, respectively. Figure 5 presents subgroup analysis results by age, showing 54 participants in the ≥19 subgroup with WMD = −0.02, 95% CI[−0.08, 0.03], Z = 0.81, and *P* = 0.42; 74 participants in the <19 subgroup with WMD = −0.1, 95% CI[−0.21, 0.00], Z = 1.88, and *P* = 0.06, indicating no significant differences between subgroups (I2 = 14.1%, *P* = 0.28) (Figure 6).

Gender subgroup analysis based on 2 male-specific and 4 female-specific articles is presented in Figure 5. In the male subgroup comprising 54 participants, WMD = −0.01 with a 95% CI[−0.08, 0.07], Z = 1.9, *P* = 0.85; while in the female subgroup (74 participants), WMD = −0.11 with a 95% CI[−0.19, −0.03], Z = 2.70, *P* = 0.007 < 0.05, suggesting the intervention was more effective in females. The comparative gender subgroup analysis showed I^2^ = 70.7%, *P* > 0.05 (Figure 5).

## Discussion

### The advantages of small-sided game training in handball

The overwhelming success of small-sided game training within the realm of football has facilitated its application to other team sports. Research indicates that the advantages of small-sided game training lie in its ability to replicate the movement patterns, skills, and physiological demands of actual competition (24), providing feedback on training effectiveness in the form of official game scenarios while allowing for timely corrections, thus ensuring strong temporal relevance (20). Training content is adjusted in accordance with in-game performance and outcomes, while also manipulating the players’ offensive and defensive areas to achieve varied training objectives, ultimately increasing the efficiency of time utilization.

When compared to conventional physical training (1) and high-intensity interval training (23), small-sided game training, through modifications to the number of players and court dimensions, increases the players’ ball contacts and extends ball possession time, improving their athletic performance and game adaptability. Mikalonytė (23) used 2v2 (20 × 10 m), 3v3 (20 × 20 m), and 4v4 (20 × 20 m) SSG formats and concluded that changes in the rules led to increased ball touches for the players and correspondingly higher defensive pressure. High-frequency technical actions, combined with the physiological workload of the games, can serve as effective strategies for honing skills (25, 26). According to Iacono (1) SSG shows significant advantages in improving lower limb strength over high-intensity interval training, possibly because players engage in more high-intensity lower limb counter-movement actions during training, which indirectly leads to a notable improvement in agility. In SSG sessions, frequent execution of hitting, blocking, carrying out offensive and defensive moves, as well as increased physical contact between players, leads to significant increases in upper body strength due to the training stimuli.

Póvoas (27) reported that during a 60-minute handball game, players covered approximately 4 kilometers at an average intensity equivalent to 87% of their maximum heart rate (HR), indicating that around 90% of the total energy expenditure is derived through aerobic activity. Mikalonytė's (23) experimental results showed that SSG, as a mid-season training strategy, not only simulated match characteristics but also developed the physical attributes of handball players, improving their sprinting, jumping, and aerobic performance. In handball games, SSG improves not only speed, agility, and upper body strength, but also enhances lower limb strength and jumping ability. For example, Dello Iacono's study on the effects of small-sided games vs. high-intensity interval training (HIIT) on handball players revealed that SSG is more effective at enhancing repeated sprint ability and jump performance (7). SSG also develops decision-making skills and teamwork, which are crucial for quick responses and team coordination in handball (23). Moreover, specific to handball, SSG increases ball contact opportunities and holding time, thereby improving players’ technical and tactical levels (1, 7). Studies indicate that SSG significantly improves handball players’ agility and standing throw ability, and also effectively boosts their speed and jump performance (11).

In summary, small-sided game training in handball is not only feasible for enhancing players’ on-field performance, upper body strength, aerobic capacity, and agility but also demonstrates that incorporating it as a training strategy during the pre-season and mid-season can effectively improve the athletic performance of handball players.

### Impact of small-sided game training on handball players’ lower limb explosive power

#### CMJ(cm)

Research (7) has suggested that similar small-sided game training positively affects agility, sprinting capacity, and jumping ability in handball, basketball, and football players. The increase in CMJ height is attributed to the synchronicity of the training regimen, improved muscle stretching efficacy, and enhanced muscle stiffness. A comparison by Pekas of small-sided game training against traditional training over a 12-week period, with three sessions per week and equivalent training loads, demonstrated that a significant portion of the improvement in CMJ was due to increased lower limb explosive power after small-sided game training (28). This also confirms the feasibility of transferring training methods between different athletic disciplines (29). Iacono posits (1) that, during SSG, the frequency of acceleration, deceleration, directional changes, jumping, defending, and braking is much higher than in HIIT. These actions involve intense muscle activity and a high degree of eccentric muscle training, potentially an effective means to boost calf muscle strength. This aligns with experiments conducted by Suraci (30). However, some scholars believe that frequent physical contact may reduce lower limb explosive power: the repeated 1v1 encounters during SSG, where players are eager to score and attempt more intense movements like jumps, might lead to a deterioration in calf muscle capacity, impeding normal lower limb power output (8).

SSG enhances CMJ performance by improving lower limb explosive power and core strength. During SSG, athletes frequently jump, change direction, and move swiftly, increasing game intensity and physical confrontation, thereby requiring strong lower limb explosive power and core stability. Studies have shown that SSG significantly improves lower limb explosive power and core strength. Additionally, dynamic stretching and lower limb strength training prior to SSG can further enhance muscle stiffness and potential energy, thus boosting CMJ performance (20). Moreover, SSG improves cardiovascular fitness, which indirectly enhances CMJ performance by providing more energy support, thus enhancing explosive power and endurance, including CMJ ability.

#### 20-m (s)

To reduce bias in these findings, a subgroup analysis was conducted on 20 m sprint capability. (1) Age. Researchers assert that during the peak of adolescent height growth (PHV), significant improvements are observed in metrics capturing explosive power, speed, agility, and neuromuscular adaptability (11, 31), making it necessary to develop these athletic traits around PHV. However, there were no significant differences for age groups <19 or ≥19 (*P* = 0.42, *P* = 0.06) indicating that age has a minimal influence on 20 m sprint capability within small-sided game training. This is consistent with the findings of Hammami (2021) (5): improvements in speed may be subject to neuronal adaptation and genetics (32). Moreover, post-SSG, the SSG group improved their 20 m sprint times by 3.91% (HIIT: 1.70%) (7). (2) Gender. Females demonstrated a greater improvement in 20 m sprint capability following small-sided game training (*P* = 0.007) with no gender differences (*P* = 0.06), suggesting women are more receptive to SSG adaptations. This aligns with the improvements seen in the preseason by the SSG group (1.68%) reported by Jurišić (22), and the enhancement (1.5%) suggested by Bělka (11), who recommended incorporating small-sided game training twice a week during the preseason to enhance female handball players’ short-distance sprint performance.

Overall, power, jumping ability, and short-distance sprinting are crucial components for success in handball (1, 5, 33). SSG incorporates high-intensity sprints, direction changes, and variable pace running, all of which require athletes to quickly engage their entire muscle strength within a short duration. This training method aids in enhancing athletes’ muscle strength and endurance, particularly in the lower limbs. For the 20-m sprint, powerful lower limb muscles are crucial for supporting rapid movement and maintaining sprinting speed. Furthermore, Adjustments to the standard training environment, such as court size (from 20 × 40 m to 10 × 10 m or others), player numbers (from the standard seven-a-side to 2v2 or other formats), and rules (with or without physical contact), alter sprint speed, jumping frequency, and foster different physical adaptations (23). Buchheit (21) notes that the frequency of acceleration and deceleration in training can offer sufficient stimulus to increase leg muscle strength. Studies confirm (7, 34) that improvements in CMJ height correlate positively with enhancements in short-distance sprinting (10 m and 20 m) capabilities, thanks to increased muscle strength after small-sided game training, enhancing knee extensors, hip extensors, and plantar flexors, culminating in augmented explosive power.

### Limitations and weaknesses of the study

The sample size used in the study may not represent all handball players, which could impact the generalizability and reliability of the results. If the study is limited to a specific age group or skill level, the findings may not be applicable to a broader athlete population. Additionally, athletes’ physiological conditions (e.g., age, gender, body composition) and psychological states (e.g., motivation, stress) may affect lower limb explosive power performance. These factors may be difficult to control entirely in study design, thus affecting the interpretation of the results. Moreover, the subgroup analysis on males included fewer studies, so the results of the gender analysis should be interpreted with caution.

## Conclusion

For 20 m sprint performance, this study found that SSG has varying impacts on handball players of different ages and genders. Specifically, there is no significant improvement in 20 m sprint performance between players aged <19 and ≥19, while female players show greater improvement in 20 m sprint compared to male players. These differences could be attributed to the physiological, psychological, and adaptive training differences between athletes of different ages and genders.

Although SSG plays an essential role in training handball players, particularly in enhancing lower limb explosive strength and overall game performance, it is recommended to combine SSG with other targeted strength and explosive power training to maximize the enhancement of lower limb explosive power in handball players. Comprehensive training can effectively improve the lower limb explosive strength of athletes while also addressing the development of other key athletic qualities to achieve the best training outcomes. Therefore, coaches should fully consider the individual differences and training needs of athletes when designing training plans, and reasonably allocate the proportion and sequence of SSG with other training methods to maximize training effectiveness.
